# Adult Pulmonary Blastoma: A Case Report with Spectrum of Rare Manifestations

**DOI:** 10.5146/tjpath.2023.01597

**Published:** 2024-01-22

**Authors:** Mayur Parkhi, Nishtha Ahuja, Divyesh Kumar, Rajender Kumar Basher, Navneet Singh, Harkant Singh, Amanjit Bal

**Affiliations:** Departments of Histopathology, Post Graduate Institute of Medical Education & Research (PGIMER), Chandigarh, India; Departments of Radiotherapy and Oncology, Post Graduate Institute of Medical Education & Research (PGIMER), Chandigarh, India; Departments of Nuclear Medicine, Post Graduate Institute of Medical Education & Research (PGIMER), Chandigarh, India; Departments of Pulmonary Medicine, Post Graduate Institute of Medical Education & Research (PGIMER), Chandigarh, India; Departments of Cardiovascular and Thoracic Surgery, Post Graduate Institute of Medical Education & Research (PGIMER), Chandigarh, India

**Keywords:** Pulmonary blastoma, Non-smoker, Immunohistochemistry, Next generation sequencing, MYCN, ATM

## Abstract

Pulmonary blastoma (PB) is an exceedingly rare and aggressive malignant lung neoplasm that has distinct biphasic morphology. In this report, we document rare manifestations and molecular alterations in PB.

A 59-year-old non-smoker female, presented with cough and hemoptysis for 4 months. The high-resolution computed tomography chest scan showed a 3.5x2.7 cm mass in the basal segment of the left lung. Positron emission tomography and computed tomography revealed a fluorodeoxyglucose avid lobulated mass in the superior segment of the lower lobe of the left lung. On core biopsy, the diagnosis of pleomorphic carcinoma in a background of adenocarcinoma was made. A definite diagnosis of pulmonary blastoma was established on the left lung lobectomy specimen based on morphological and immunohistochemical findings. Post-surgical biopsy from the scalp swelling showed metastatic deposits. On Next Generation Sequencing (NGS), in addition to conventional *CTNNB1* gene mutation, new pathogenic *MYCN* and *ATM* gene mutations were detected. Post-chemotherapy, the patient was doing well after 10 months of close follow-up.

PB exhibited rare associations in the form of non-smoker status, scalp metastasis, and *MYCN* and *ATM* gene mutations on *NGS* in addition to conventional *CTNNB1* gene mutation. Large cohort studies are required to discover the incidence, significance and therapeutic implications of these co-existing pathogenic molecular alterations in PB.

## INTRODUCTION

Pulmonary blastoma (PB) is an exceedingly unusual and aggressive malignant neoplasm of the lung that accounts for 0.25% to 0.5% of all resected lung cancers (1). It principally consists of immature mesenchymal and epithelial elements that structurally mimic the embryonic lung. In 1945, Barrett and Bernard were the first ones to bring forward this entity; and Bernard later called it ‘embryoma’ (2,3). A decade later, Spencer termed this neoplasm as pulmonary blastema based on its histological resemblance to the fetal lung (4). Despite this embryonic derivation, it commonly affects the adult population rather than childhood. As per the recent 2021 world health organization (WHO) classification of thoracic tumours, this rare neoplasm along with pleomorphic carcinoma and carcinosarcoma comes under the broad category of sarcomatoid carcinomas (5). Till date, the most common genetic alteration is the missense mutation in exon 3 of the *CTNNB1* gene (6). The survival outcome in these patients is very poor due to the high rate of tumour recurrence and distant metastasis, and about two-third of the patients succumb to the disease within 2 years of the diagnosis (7). Herein, we present a non-smoker female in her late adulthood diagnosed with PB showing scalp metastasis and unique molecular alterations on next-generation sequencing (NGS).

## CASE REPORT

### Clinical Presentation

**Figure 1:**
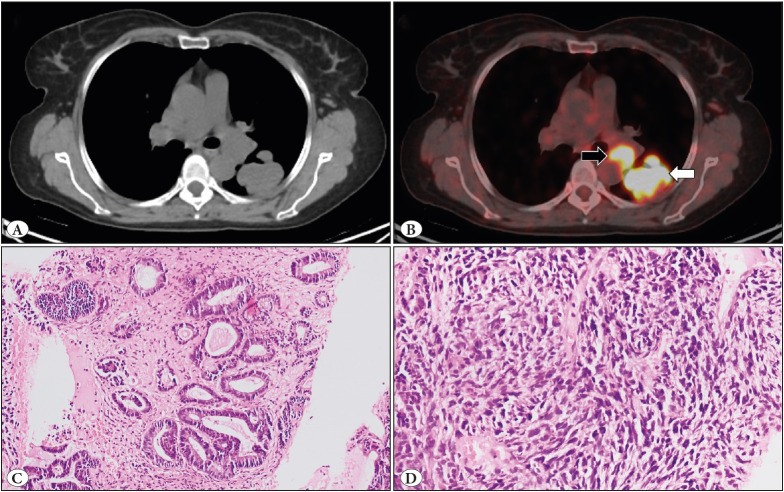
**A,B**) An intensely FDG avid lobulated soft tissue lesion (white arrow) in the superior segment of the left lung lower lobe with an FDG avid enlarged left hilar lymph node (black arrow) on the trans-axial CT and fused PET-CT imaging. **C**) The epithelial component displays complex acinar and glandular growth pattern with moderately pleomorphic nuclei (H&E; x200). **D**) The mesenchymal component appeared oval-to-spindle shaped, and displayed a mild degree of pleomorphism, hyperchromatic nuclei, and inconspicuous nucleoli (H&E; x200).

A 59-year-old female, a non-smoker, presented with complaints of productive cough and hemoptysis for 4 months. In association, loss of weight and appetite was also noted; however, there was no history of fever, abdominal pain, or distension. Fifteen years back, she was operated for haemorrhoids. The high-resolution computed tomography (HRCT) chest scan showed a 3.5x2.7 cm soft tissue mass with smooth margins in the basal segment of the left lung. Computed tomography (CT) and positron emission tomography and computed tomography (PET-CT) scan of the whole body revealed a fluorodeoxyglucose (FDG) avid lobulated soft tissue mass in the superior segment of the lower lobe of the left lung (Figure 1). In addition, FDG avid necrotic enlarged left hilar lymph node and FDG avid circumferential wall thickening involving the rectum and anal canal, and the perilesional mesorectal and presacral lymph nodes were also noted. Considering the imaging findings, lower gastrointestinal (GI) bleeding was evaluated first with suspected primary or metastatic rectal malignancy. However, lower GI endoscopy guided biopsy did not reveal any evidence of malignancy. Thus, we received the following samples in a sequential manner: core left lung biopsy and left lower lobectomy specimen.

### Histopathological aspects

The biopsy from the left lung mass revealed a cellular tumour with mainly epithelial (~90%) and some mesenchymal (~10%) component. The epithelial component showed complex acinar and glandular growth patterns (Figure 1). The tumour cells were moderately pleomorphic with round nuclei, dispersed chromatin, inconspicuous nucleoli and moderate eosinophilic cytoplasm. The mesenchymal component appeared oval-to-spindle shaped, and displayed a mild degree of pleomorphism, hyperchromatic nuclei, and inconspicuous nucleoli (Figure 1). Areas of necrosis were also noted. The diagnosis of pleomorphic carcinoma against the background of adenocarcinoma was suggested.

**Figure 2:**
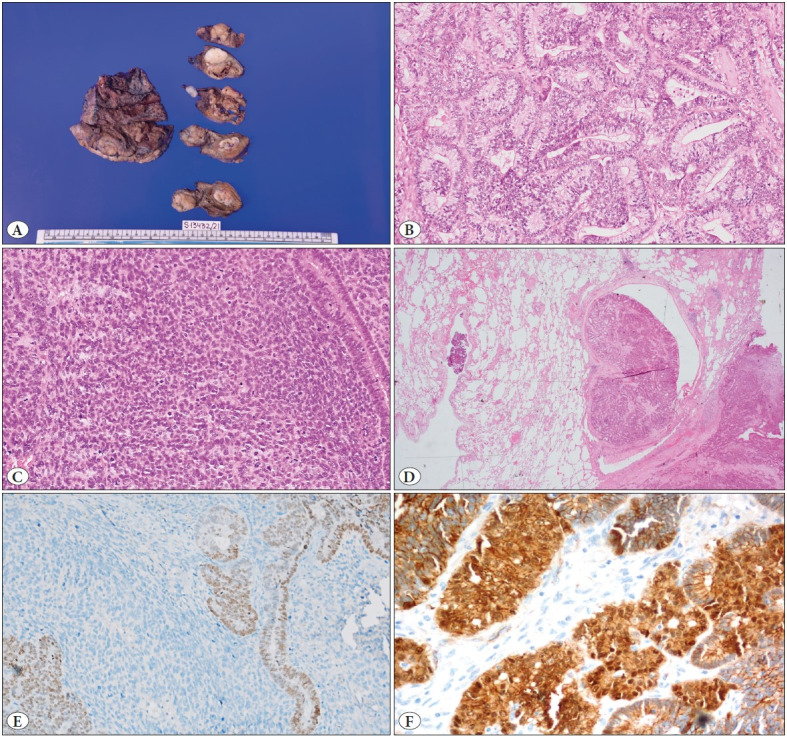
**A**) On serial slicing, the left lower lobectomy specimen showing a solitary, ill-defined, grey-white, firm tumour mass. **B**) The epithelial cells, arranged predominantly as elongated glands, show nuclear pseudo-stratification and overcrowding with subnuclear vacuolation (H&E; x200). **C**) The primitive mesenchymal cells exhibit high cellularity, moderately pleomorphic nuclei, and brisk mitotic figures (H&E; x200). **D**) Lymphovascular space emboli is seen (H&E; x20). **E**) Epithelial component expressing TTF-1 immunostain (Peroxidase; x100). **F**) beta-catenin showing nuclear and cytoplasmic positivity in the epithelial cells (Peroxidase; x200).

One month later, the left lower lobectomy specimen measuring 14x6x2.5 cm was received. On serial slicing, a solitary, ill-defined, grey-white, firm tumor mass was seen measuring 5x3x3 cm (Figure 2). The tumor was not infiltrating the hilar vessels and lobar bronchus. The rest of the lung parenchyma was sub-crepitant to feel. One hilar lymph node measuring 0.5 cm in diameter was grossly involved by tumor. Lymph nodes (station 3, 4, 5, 6, 7, 8, 9, 10) were also submitted along with the main specimen. Microscopically, the tumor had biphasic morphology that contained malignant epithelial and primitive mesenchymal components. Both these components were mainly discrete but intermingled with each other in some areas. The epithelial cells were arranged predominantly as elongated glands and few discrete foci of solid sheet and cribriform growth patterns (Figure 2). The glands displayed nuclear pseudo-stratification and overcrowding with subnuclear vacuolation. The mild to moderately pleomorphic tumor cells had round to oval nuclei, vesicular to dispersed chromatin, inconspicuous to small distinct nucleoli, and moderate clear to eosinophilic cytoplasm. Mitotic activity was intermediate to high (8-12/10 high power fields). Squamous morules were also identified. The primitive mesenchymal cells were highly cellular and moderately pleomorphic (Figure 2). The nuclei were oval-to-spindle with clear to dispersed chromatin, inconspicuous nucleoli, and scant cytoplasm. Mitoses was atypical and brisk (>15 per 10 high power fields). Areas of necrosis and hemorrhage were also noted but no heterologous elements were seen. Lymphovascular emboli and visceral pleural invasion was present (Figure 2). No spread through air space was noted. On immunohistochemistry (IHC), the epithelial component showed membranous and nuclear expression for pan-cytokeratin (Dako, clone MNF116, dilution 1:100) and TTF-1 (Cell Marque, clone 8G7G3/1, dilution 1:300), respectively (Figure 2). The cytoplasmic and nuclear expression of beta-catenin (Cell Marque, clone 14, dilution 1:100) was noted in glandular component (Figure 2). Chromogranin (Dako, clone DAK-A3, dilution 1:100) and synaptophysin (Cell Marque, polyclonal, dilution 1:150) were negative and the ki-67 (Cell Marque, clone SP6, dilution 1:300) proliferating index was around 80%. The hilar lymph node showed metastatic tumor deposits with no extranodal extension; however, the remaining station lymph nodes were uninvolved. Surgical margins including bronchus, vascular and parenchymal were free. The diagnosis of pulmonary blastoma, pT2bN1 (American Joint Committee on Cancer (AJCC) Staging Manual; Eighth Edition) was rendered.

**Figure 3:**
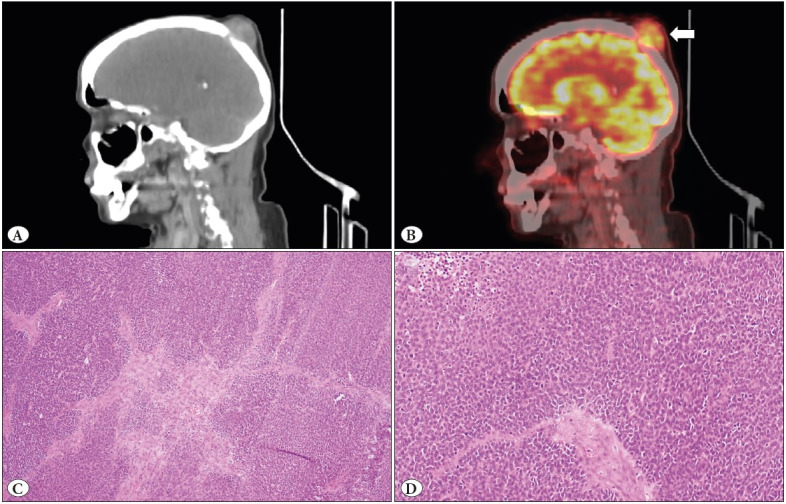
**A,B**) The coronal reformatted CT and PET-CT images of the head shows an FDG avid soft tissue mass in the scalp with lytic destructive changes in the skull and minimal intracranial extension. **C**) The scalp metastasis contains only the mesenchymal element in variable sized nodules and islands with similar morphology as primary lung tumor (H&E; x100). No epithelial element is identified. **D**) Brisk mitotic activity is visible (H&E; x400).

Two months after lobectomy, the patient developed a scalp lesion which showed an FDG avid uptake with lytic destructive changes in the skull and minimal intracranial extension on the coronal reformatted CT and PET-CT images of the head (Figure 3). A wide local excision specimen measuring 6x4.5x1cm was sent. On serial slicing, there was a partly demarcated grey-white and firm mass measuring 5.2x4x0.7cm. Microscopically, the tumor showed only mesenchymal component with primitive cells arranged in variable-sized nodules and islands (Figure 3). No epithelial element was identified even after thorough tumour tissue sampling. The morphology and IHC findings were similar to lung mass. Along with this specimen, there was a bone biopsy that also showed infiltration by tumor. The diagnosis of metastatic PB was given (pM1b).

### Next Generation Sequencing

The DNA isolated from paraffin-fixed formalin-embedded tissue was subjected for Next-Generation Sequencing and it revealed mutations in the β-catenin (*CTNNB1*) gene c.98C>G (ENST00000645 320.1) p.Ser33Cys/Exon 3 gain of function mutation, *MYCN* gene c.131C>T (ENST00000281 043.4) p.Pro44Leu / Exon 2 gain of function mutation, and *ATM* gene c.5631_5635deli nsA (ENST00000278 616.8) p.Phe1877Leufs Ter39 / Exon 37 loss of function deletion.

### Treatment and Follow-Up

The patient received one cycle each of neoadjuvant and adjuvant chemotherapy before and after surgery, respectively. The chemotherapy agents include pemetrexed, carboplatin, and etoposide. In addition, the patient received radiotherapy with 8 Gy per fraction for the scalp metastatic site. The patient is alive till last 11 months of close follow-up; but recently he was detected with metastasis in the right paravertebral region and subcarinal lymph node on positron emission tomography computed tomography (PET-CT).

## DISCUSSION

PB is one of the rare lung cancers that show biphasic morphology containing fetal adenocarcinoma (low grade or well-differentiated) and primitive mesenchymal stroma (5). Pulmonary blastoma is considered as a separate entity under the broad category of sarcomatoid carcinoma as per the recent WHO classification of thoracic tumors (fifth edition; 2021) (5). Thus, it has the distinct biphasic morphology, and aggressive behavior with a 5-year survival rate of 16% (5,7).

On computed tomography, the tumor appears well-circumscribed with varying contrast uptake and central necrosis (8). The definite diagnosis comes from the histopathological examination of the surgical specimens. The common differentials considered are WDFA, carcinosarcoma, and pleomorphic carcinoma. These differentials are important because PB has poor prognosis compared to WDFA and better prognosis compared to carcinosarcoma. There are limitations of diagnosing these lesions on a core biopsy due to caveats like pure sampling of either the epithelial or heterologous elements which will create challenges for the pathologists that may affect treatment decision. The tumor showing pure epithelial component, absence of mesenchymal element and consistent lack for *TP53* mutation favor WDFA (9). The possibility of carcinosarcoma can be kept when the tumor contains high-grade fetal-type or clear cell adenocarcinoma with heterologous elements, lacks squamous morules, membranous expression of beta-catenin and the molecular features of blastoma (5). Pleomorphic carcinomas are defined by the presence of non-small cell lung carcinoma with at least 10% of the tumor area showing spindle cells and/or giant cells (5). Considering these pathological criteria, the diagnosis of pleomorphic carcinoma which we made on core biopsy was later changed to PB on surgical specimen.

There is limited knowledge about the pathogenesis of PB. Studies have shown strong correlation with the smoking status. At molecular level, the most commonly associated genetic alteration is a missense mutation in exon 3 of the *CTNNB1* gene which leads to WNT pathway activation leading to nuclear localization of beta-catenin (6). However, this mutation is not specific to PB cases and may be detected in cases of WDFA and less often in PPB cases (5). Another common mutation detected in PB is *TP53* mutation which may also be found in PPB but not in WDFA cases (10). Besides these two mutations, the literature also showed molecular alterations involving *ROS1*, *EGFR*, *BRCA2*, *ERBB4*, *ALK*, *MET*, *BRAF*, *RAF1*, *PTEN*, and *PIK3CA* genes (11,12). Additionally, the somatic *DICER1* mutations coupled with *CTNNB1 *mutations was identified in two cases of adult-onset pulmonary blastoma (13). Thus, a potential genetic link to pediatric pleuropulmonary blastoma was indicated. Recently, *MYCN* mutation was detected in the micro dissected mesenchymal component based on capture-based targeted next generation sequencing method (14). In the index case, we came across a pathogenic mutation in the *MYCN* and *ATM* genes on NGS where the latter have not been described in the literature as per our best knowledge. Terra et al. (2016) analyzed 33 cases of pulmonary sarcomatoid carcinoma for approximately 2800 mutations in 50 oncogenes and tumor-suppressor genes on NGS (15). They did not detect any case showing *MYCN* and *ATM* gene mutation. Though the role of these mutations has not yet been fully discovered in adult pulmonary blastoma but it is known to be associated with poor prognosis and distant metastasis in other malignancies (e.g., neuroblastoma, breast cancer) (16,17). It is documented that the microRNA-421 mediates downregulation of *ATM* gene via overexpression of *MYCN* transcriptional factor in neuroblastoma; however, this relation in pulmonary blastoma needs to be discovered through large cohort studies (18).

The PBs have the tendency of both local extensions into adjacent structures and distant hematogenous spread. The brain, bones, liver, breast, peritoneum and ovaries are the common metastatic sites documented (8). Very few cases of PB with cutaneous spread, including the index case, are reported (19). The treatment in the form of surgery, and chemotherapy (neoadjuvant and adjuvant), and/or radiotherapy are considered depending on the tumour stage. Surgical resection (i.e., lobectomy) is mainly done as most of the tumours are detected in the early stage. When it comes to metastasis, there are no specific management guidelines available (9).

In conclusion, pulmonary blastoma is an exceedingly rare lung cancer that shows distinct biphasic morphology. The definite diagnosis can be made on surgical specimens as core biopsies have their limitations. In the index case, this neoplasm exhibited rare associations in the form of non-smoker status, scalp metastasis (cutaneous spread), and pathogenic molecular alterations in the *MYCN* and *ATM* genes on NGS in addition to conventional *CTNNB1* gene mutation. In view of these co-existing pathogenic molecular alterations, large cohort molecular studies are required to discover the incidence, significance, and therapeutic implications in pulmonary blastoma.

## Conflict of Interest

The authors declare that there is no conflict of interest.

## Funding

The authors have no funding or financial relationships to disclose.

## Informed Consent

Informed written consent was taken from the patient.
